# Ear Surgery Training: A Comprehensive Review

**DOI:** 10.7759/cureus.109175

**Published:** 2026-05-19

**Authors:** Mara Tanase, Mihai I Tănase, Marcel Cosgarea, Alma A Maniu, Doinel G Rădeanu, Mirela C Stamate, Constantin Stan, Cristina Blebea, Maximilian Dindelegan, Violeta Necula

**Affiliations:** 1 Department of Otolaryngology, ”Iuliu Haţieganu” University of Medicine and Pharmacy, Cluj-Napoca, ROU; 2 Department of ENT, University of Alba Iulia "1 Decembrie 1918", Alba Iulia, ROU; 3 Department of ENT, ”Iuliu Haţieganu” University of Medicine and Pharmacy, Cluj-Napoca, ROU; 4 Research Centre in the Medical-Pharmaceutical Field, Faculty of Medicine and Pharmacy, “Dunarea de Jos” University of Galati, Galati, ROU; 5 Department of Surgery - Practical Abilities, "Iuliu Hatieganu" University of Medicine and Pharmacy, Cluj-Napoca, ROU

**Keywords:** 4k-3d exoscope, haptic trainer, otolaringology, skill training, virtual reality (vr)

## Abstract

The technical complexity of otologic procedures, such as mastoidectomy and ossiculoplasty, necessitates high-fidelity training to navigate intricate temporal bone anatomy safely. This review evaluates the transition from traditional surgical pedagogy to high-tech simulation. We synthesized current data on cadaveric dissection, 3D-printed models, and virtual reality (VR) platforms, assessing their impact on haptic proficiency and clinical skill transfer. Beyond traditional methods, we examine how exoscopic and robotic-assisted systems are reshaping the learning curve by providing enhanced magnification and tremor filtration. However, the integration of these tools faces hurdles, including high capital costs and the challenge of replicating the varied pathologies encountered in live patients. Our analysis highlights that while emerging technologies offer scalable alternatives to human tissue, traditional mentorship remains the benchmark for haptic feedback. A comprehensive, multimodal training framework is essential to bridge the gap between simulation and the operating room, ultimately optimizing patient safety and surgical precision.

## Introduction and background

The technical complexity of otologic procedures -- including mastoidectomy, tympanoplasty, and ossiculoplasty -- presents unique challenges due to the intricate temporal bone anatomy and the proximity of vital neurovascular structures. Traditionally, surgical training has centered on cadaveric temporal bone dissection and mentorship, providing trainees with invaluable tactile experience and a deep understanding of spatial relationships. However, the field is evolving to incorporate modern modalities such as virtual reality (VR) simulation and 3D-printed models, which offer safe, repeatable environments for skill acquisition without the limitations of specimen availability. Emerging technologies, including exoscopic and robotic systems, further refine the learning curve by enhancing magnification and dexterity. This systematic review evaluates these diverse training modalities, assessing their anatomical accuracy, haptic feedback, and cost-effectiveness to define a multimodal framework for modern surgical pedagogy [1].

Ear surgery, an intricate surgical specialty, demands exceptional precision and expertise due to the ear's complex anatomy and the presence of numerous vital structures. The successful outcome of ear surgery is highly dependent on the surgeon's skill and understanding of this anatomical region. Traditionally, ear surgery training has centered around cadaveric temporal bone dissection, providing aspiring surgeons with invaluable hands-on experience and a profound understanding of the ear's complex anatomy. This method allows for the development of essential surgical skills, such as drilling, dissecting, and suturing, in a safe and controlled environment [2].

Modern ear surgery training has evolved to incorporate various approaches, including VR simulation and 3D-printed models. VR simulators offer a safe and repeatable environment for practicing surgical techniques, enhancing depth perception and spatial orientation. These simulators can be customized to replicate different ear pathologies and surgical scenarios, providing a comprehensive training experience. Three-dimensional-printed models offer a tangible and realistic representation of the ear's anatomy, allowing surgeons to practice surgical procedures and improve their understanding of spatial relationships [3].

In addition to the training methods mentioned above, ear surgery training also encompasses mentorship and hands-on experience in the operating room. Mentorship provides guidance and feedback from experienced surgeons, while hands-on experience allows trainees to apply their knowledge and skills in a real-world setting. As technology advances, ear surgery training is likely to incorporate more innovative methods, such as exoscopic and robotic surgery. Exoscopic surgery utilizes a high-definition camera to provide a magnified view of the surgical field, enhancing precision and ergonomics. Robotic surgery, while still in its early stages, offers the potential for minimally invasive procedures with increased dexterity and precision [4].

The choice of training method depends on various factors, including cost, accessibility, and the specific skills being taught. Cadaveric dissection remains the gold standard for developing haptic feedback and anatomical understanding. However, VR simulation and 3D-printed models offer valuable alternatives, particularly for repetitive practice and the development of specific surgical skills [5].

Despite the numerous advantages, there are some limitations to consider. The texture and consistency of 3D-printed models may not perfectly replicate real tissue, which could affect the tactile feedback during simulated procedures. Additionally, the accuracy of the models depends on the quality of the imaging data used for their creation [6].

The application of VR simulation in ear surgery training is still in its early stages, but the potential benefits are significant. As technology continues to evolve, we can expect even more realistic and sophisticated simulations that will further enhance the training experience and improve surgical outcomes [7].

## Review

Methods

**Figure 1:**
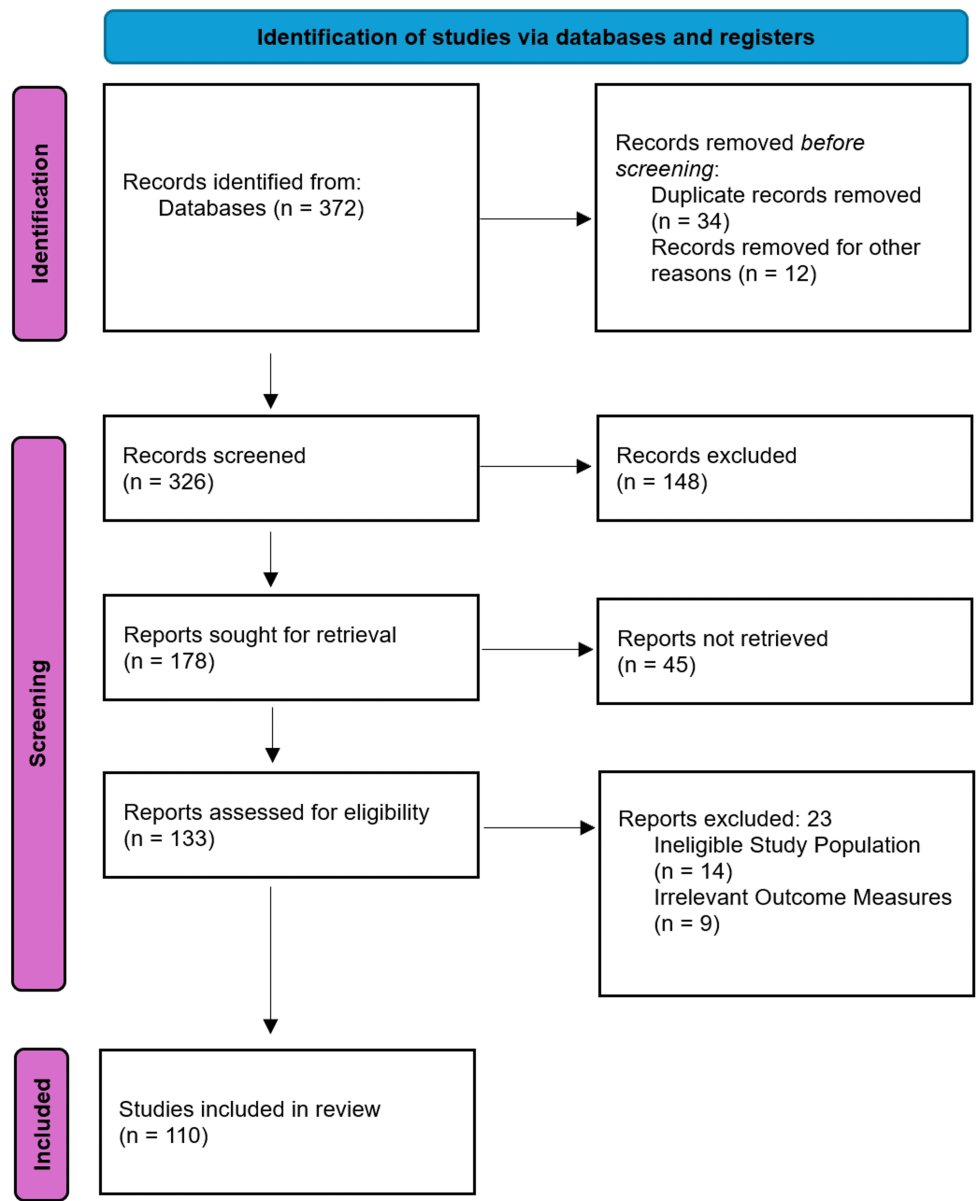
PRISMA Flowchart

This study was conducted as a systematic review to evaluate the current landscape of ear surgery training modalities. A comprehensive literature search was performed across the PubMed, Scopus, and Web of Science databases, utilizing search terms including "otologic surgery training," "temporal bone simulation," "virtual reality in ENT," and "3D printing mastoidectomy." The search was limited to peer-reviewed articles published in English between 2000 and 2026 to ensure the inclusion of recent technological advancements. Following the PRISMA framework (Figure 1), an initial pool of 372 records was identified. After removing duplicates and screening for relevance to surgical pedagogy, 133 reports were assessed for eligibility. Ultimately, 110 studies were included based on their focus on anatomical accuracy, haptic feedback, cost-effectiveness, and clinical skill transfer. Studies involving ineligible populations or irrelevant outcome measures were excluded to maintain a focused analysis of training efficacy.

Review

Challenges in ear surgery

Ear surgery, also known as otologic surgery, focuses on the surgical treatment of ear diseases and disorders. Due to the intricate anatomy of the ear and its proximity to critical structures like the facial nerve, inner ear, and brain, ear surgery presents unique challenges, demanding specialized training and expertise from ear surgeons to ensure successful outcomes and minimize complications [8].

The ear comprises three main parts: the outer ear, middle ear, and inner ear, each with unique anatomical structures and functions. Ear surgeons must have a thorough understanding of these structures to perform successful surgeries. The middle ear houses the ossicles, the smallest bones in the human body, responsible for transmitting sound vibrations to the inner ear. The inner ear contains the cochlea, a spiral-shaped structure that converts sound vibrations into electrical signals sent to the brain [9].

One of the primary challenges in ear surgery is preserving the ear's function, particularly hearing and balance. The structures of the inner ear, responsible for hearing and balance, are highly susceptible to damage during surgery. Ear surgeons must exercise extreme caution and precision to avoid causing any functional impairment [10].

The field of ear surgery has witnessed significant technological advancements, including new surgical tools and techniques, leading to improved surgical outcomes and reduced complication rates. However, these advancements also pose challenges for ear surgeons, who must stay abreast of the latest developments and acquire the necessary skills to utilize these new technologies effectively [11].

Preserving the ear's function, especially hearing and balance, is a significant challenge. Hearing depends on the elaborated interplay of various structures within the ear. Sound waves are collected by the pinna, travel through the ear canal to reach the tympanic membrane (TM), and the vibrations are transmitted through the ossicles to the cochlea. Inside the cochlea, these vibrations stimulate hair cells that convert the mechanical energy into electrical signals, which are then sent to the brain via the auditory nerve [12].

Balance is maintained by the vestibular system in the inner ear, consisting of three semicircular canals and two otolith organs, which detect head movements and provide information to the brain about the body's position and orientation. Any damage to these internal structures during surgery can result in hearing loss, tinnitus, or balance problems like vertigo. Therefore, it is important to exercise extreme caution and precision to avoid any inadvertent injury to these critical structures [13].

Anatomical Complexity and Functional Preservation

The surgical management of otologic disease is defined by high anatomical density and the proximity of vital neurovascular structures within the temporal bone [14,15]. Mastery of this compartmentalized architecture, spanning the external auditory canal (EAC), the ossicular chain, and the sensorineural components of the inner ear, is the fundamental prerequisite for achieving therapeutic goals while minimizing iatrogenic trauma [16-18]. Clinically, it is essential for trainees to distinguish between the meatus (the visible lateral opening) and the EAC (the full tube leading to the TM) to ensure precise instrument orientation [19,20].

A primary challenge in ear surgery is the preservation of hearing and balance, as the structures responsible for these senses are highly susceptible to surgical injury [21]. Hearing relies on the elaborate interplay between the TM, the ossicles, and the hair cells of the cochlea [22]. Simultaneously, balance is maintained by the vestibular system, where the semicircular canals and otolith organs act as primary sensors. High-fidelity training must emphasize the role of the vestibulospinal reflex (VSR) and vestibulo-ocular reflex (VOR), which translate head movements into motor responses, as these systems are at risk during complex neurotologic procedures [23,24].

Preserving these functions requires extreme caution and precision, particularly during procedures like mastoidectomy or stapedotomy. The proximity of the facial nerve to the surgical field, specifically near the oval window and the mastoid air cells, represents a significant risk for iatrogenic injury, which can result in permanent facial paralysis [25,26]. Consequently, surgical pedagogy must utilize a combination of anatomical study and simulated practice to prepare trainees for the high-stakes environment of the operating suite, where anatomical variations such as a high-riding jugular bulb or an exposed facial nerve may be encountered [27,28].

Technological Advancements in Training

The field of ear surgery has witnessed remarkable technological growth, offering new possibilities for improved patient care through enhanced precision [29]. However, the integration of these advancements into standardized training curricula presents significant challenges, particularly regarding high capital costs and the necessity for continuous adaptation. Rather than replacing traditional training, these technologies should be used to standardize exposure, quantify progression, and identify performance gaps before supervised operating-room participation [30].

Exoscopic systems have emerged as a significant advancement, providing a shared high-definition view for trainees and improving surgical ergonomics by reducing neck strain [31]. However, their implementation requires a distinct learning curve and may induce visual fatigue or eye strain during prolonged use. While they facilitate a deeper understanding of anatomical relationships, trainees must familiarize themselves with the technology to optimize its benefits in a clinical setting [32].

Similarly, VR simulators and 3D-printed models allow for risk-free, repetitive practice of complex procedures such as mastoidectomies and stapedotomies [33]. These high-fidelity models enable surgeons to anticipate anatomical challenges and reduce operative time in a risk-free environment. Despite these benefits, a persistent "haptic gap" remains, as digital simulations often fail to perfectly replicate the nuanced tactile resistance and bone-drilling characteristics of live biological tissue [34].

Ultimately, the future of otologic surgical education relies on a multimodal framework that integrates simulation, augmented reality, and AI-assisted assessment with structured mentorship. Such a comprehensive approach ensures that while simulators refine manual dexterity, the irreplaceable role of expert clinical judgment is preserved. This integration is essential to bridge the gap between simulation and the operating room, optimizing both patient safety and surgical precision [35].

Types of ear surgery

Otologic surgery constitutes a multifaceted discipline requiring both exhaustive anatomical knowledge and microscopic precision. The surgical spectrum is remarkably diverse, ranging from reconstructive procedures for hearing restoration and the management of chronic inflammatory pathologies to the complex excision of skull-base tumors and the correction of congenital aural atresia. This clinical breadth necessitates a versatile surgical skillset capable of addressing both functional impairment and life-threatening disease [36,37].

Mastoidectomy and Complications

Mastoidectomy focuses on the mastoid part of the temporal bone, which forms the posteroinferior portion of the skull base just behind the auricle [38]. The procedure is necessitated by chronic infections or cholesteatoma that invade the honeycomb-like network of air cells within the bone [39]. This surgery carries a variable complication profile depending on the pathology and patient factors, with overall complication rates ranging from 10% to 45%. Radical mastoidectomy typically carries the highest risk of adverse events [40].

The proximity of the facial nerve is the most significant challenge, as injury to this nerve during bone removal can lead to permanent paralysis. To mitigate this, intraoperative facial nerve monitoring is used to locate and protect the nerve during the procedure [41]. High-resolution CT scan remains the undisputed gold standard for pre-operative planning, providing a mandatory roadmap of the temporal bone's pneumatization and facial nerve topography [42].

Tympanoplasty and Myringoplasty

Tympanoplasty is categorized by the extent of middle ear reconstruction required to repair a perforated TM [43]. Type 1 tympanoplasty, often more accurately referred to as myringoplasty, is limited to repairing the TM when the ossicular chain is intact. The surgical goal is to restore the eardrum's integrity and vibration capability to improve conductive hearing [44].

The procedure typically involves raising a tympanomeatal flap (TMF) through radial and circumferential incisions lateral to the tympanic annulus [45]. The edges of the perforation are meticulously freshened, rather than simply elevated, to prepare the tissue for grafting. Surgeons employ either an onlay or underlay technique, positioning the graft -- often autologous fascia or cartilage -- to bridge the defect and ensure proper healing [46].

Stapes Surgery: Stapedotomy vs. Stapedectomy

Stapes surgery is the primary intervention for otosclerosis, a condition where the stapes bone becomes fixed and prevents sound transmission to the inner ear [47]. While stapedectomy is the older technique involving the removal of the entire footplate, modern surgical pedagogy emphasizes stapedotomy as the current gold standard. This transition reflects a shift toward more conservative, precise interventions in otologic surgery [48].

In a stapedotomy, a small calibrated fenestra (approximately 0.6-0.8 mm) is created in the footplate using a microdrill or laser to house a piston prosthesis [49]. This approach is preferred because it offers superior high-frequency hearing gains and carries a lower risk of severe complications, such as sensorineural hearing loss or perilymph gushers. Proper placement of the prosthesis is critical to avoid displacement, which can compromise long-term hearing outcomes [50].

Ossiculoplasty

Ossiculoplasty aims to reconstruct the ossicular chain using prosthetics or autologous tissue to restore efficient sound transmission to the oval window [51]. This surgery requires high dexterity, as the surgeon works with the smallest bones in the human body within a constrained surgical field. Success is contingent upon the meticulous placement of the prosthesis to avoid extrusion or displacement [52].

One of the main challenges is achieving optimal hearing outcomes. Factors such as the extent of ossicular damage, the type of prosthesis used, and the condition of the middle ear structures can all influence the final hearing result [53,54]. 

Ossiculoplasty also carries the risk of complications, including prosthesis displacement, extrusion, or failure, as well as persistent or recurrent conductive hearing loss. Careful surgical technique, appropriate prosthesis selection, and careful postoperative care are essential to minimize these risks [55]. 

Cochlear Implantation

Cochlear implantation is a surgical procedure that involves implanting a small electronic device into the inner ear to provide a sense of sound to individuals with severe to profound sensorineural hearing loss. This procedure is often recommended for those who do not benefit from traditional hearing aids [56]. 

The cochlear implant has two main components: an external portion that sits behind the ear and an internal portion that is surgically placed under the skin. The external portion contains a microphone, a speech processor, and a transmitter. The internal portion consists of a receiver and an electrode array [57]. 

Access to the middle ear is achieved via a postauricular incision, followed by a cortical mastoidectomy to expose the antrum. A posterior tympanotomy is then performed through the facial recess to provide a direct line of sight to the round window or a cochleostomy site. The multi-channel electrode array is subsequently threaded into the scala tympani of the cochlea, facilitating direct electrical stimulation of the remaining spiral ganglion neurons [58]. 

The electrode array stimulates the auditory nerve, bypassing the damaged hair cells, and sends electrical signals to the brain, which are interpreted as sound. The external portion of the implant captures sound waves and transmits them to the internal receiver, which then sends signals to the electrode array [59]. 

Cochlear implantation is a technically demanding microsurgical procedure where the margin for error is minimized by the proximity of inner ear architecture. Achieving optimal electrode placement while preserving intracochlear structures requires a high degree of surgical precision and specialized neurotologic expertise. To attain this level of proficiency, rigorous training on high-fidelity models, such as 3D-printed temporal bones and virtual reality (VR) simulators, is essential for mastering the spatial constraints of the facial recess and cochleostomy. While the procedure serves as a transformative intervention for severe-to-profound sensorineural hearing loss, it functions as a sensory bypass rather than a biological cure, requiring extensive neural plasticity to interpret the synthesized signal. Ultimately, clinical success is multifactorial, contingent not only on the atraumatic execution of surgery but also on patient-specific variables such as age at implantation and duration of auditory deprivation [60,61].

Training modalities for ear surgery

Training modalities for ear surgery have evolved significantly, encompassing a range of approaches to equip surgeons with the necessary skills and knowledge. Traditional methods, such as cadaveric temporal bone dissection and mentorship, provide hands-on experience and anatomical understanding. However, with advancements in technology, modern approaches like VR simulation and 3D-printed models offer a safe and repeatable environment for practicing surgical techniques and refining dexterity. These modern methods allow surgeons to hone their skills without the limitations of cadaver availability or the pressures of the operating room. As technology continues to advance, emerging technologies like exoscopic and robotic surgery are likely to play an increasingly important role in ear surgery training, further enhancing precision and minimizing invasiveness [62].

Traditional Methods

Traditional methods of training in ear surgery have long relied on two key approaches: cadaveric temporal bone dissection and mentorship. These time-tested methods provide a foundation for understanding the complexities of ear anatomy and surgical techniques. Cadaveric temporal bone dissection offers a unique opportunity for surgeons to explore the intricate anatomy of the ear in a hands-on, 3D manner. Working with real specimens allows trainees to develop a deeper appreciation for the spatial relationships between various structures, including the ossicles, facial nerve, and inner ear components. The tactile feedback gained from dissecting cadaveric bones helps surgeons refine their dexterity and precision. However, the availability of cadaveric temporal bones can be limited, and ethical considerations must be considered. Due to these limitations, animal models, such as sheep temporal bones, have been explored as alternatives for surgical training due to their anatomical similarities to human temporal bones. They offer a valuable opportunity for surgeons to practice surgical techniques and gain experience in a setting that resembles human anatomy. Mentorship also plays a crucial role in ear surgery training, providing guidance and support from experienced surgeons. Mentors offer valuable insights into surgical techniques, decision-making, and patient management. They observe trainees during procedures, provide constructive feedback, and help them navigate the challenges of ear surgery. The one-on-one interaction with a mentor allows for personalized instruction and fosters the development of surgical judgment and confidence [62,63].

Cadaveric dissection: For generations, cadaveric temporal bone dissection has been the cornerstone of ear surgery training, providing an unparalleled tactile and 3D exploration of human anatomy [64]. By working with real specimens, trainees gain a deep understanding of the spatial relationships between the ossicles, facial nerve, and inner ear components. This hands-on approach allows for the refinement of drilling and dissecting skills in a safe environment, building the dexterity required before operating on live patients [65].

However, this "gold standard" faces significant logistical and financial hurdles. A whole-body cadaver can cost up to $5,000 in the United States, with single-session surgical simulation costs for a temporal bone reaching approximately $2,550. While coordinated multi-department reuse can lower this to $1,375, the substantial institutional budgeting required remains a barrier compared to animal models (~$550 per use) [66]. Additionally, cadaveric specimens lack dynamic physiological responses like bleeding and are subject to complex ethical and religious considerations regarding the use of human tissue [67].

Animal models: Animal models have emerged as valuable adjuncts to cadaveric dissection in ear surgery training. While human cadaveric temporal bones remain the gold standard, the limited availability and ethical considerations associated with their use have prompted the exploration of animal models as alternatives [68]. 

Various animal species, including sheep, pigs, and goats, have been used for temporal bone dissection training. Some studies showed that even rats can be used for otologic research or some steps of surgical procedures, their anatomy being similar to the human one [69]. The choice of animal model depends on several factors, such as anatomical similarity to the human ear, cost-effectiveness, and ethical considerations.

Sheep temporal bones, in particular, have gained popularity due to their anatomical similarities to human temporal bones. They offer a valuable opportunity for surgeons to practice surgical techniques and gain experience in a setting that closely resembles human anatomy [70]. 

Animal models provide several advantages in ear surgery training. They offer a more readily available and cost-effective alternative to human cadaveric specimens. Additionally, the use of animal models allows for a wider range of training scenarios, including the simulation of specific ear pathologies and surgical techniques [71].

However, it is essential to acknowledge that animal models are not perfect replicas of human anatomy. There are anatomical differences between species that trainees must be aware of. Despite these limitations, animal models provide a valuable adjunct to cadaveric dissection, offering a more accessible and versatile platform for surgical training [72].

Mentorship: Mentorship is a cornerstone of ear surgery training, providing aspiring surgeons with invaluable guidance and support from experienced practitioners. The relationship between a mentor and mentee fosters a dynamic learning environment where knowledge and skills are passed down through generations of surgeons [73]. 

Mentors offer more than just technical expertise; they provide insights into the art of surgical decision-making, patient communication, and ethical considerations. They help trainees navigate the complexities of ear surgery, offering advice on patient selection, surgical planning, and postoperative care [74]. 

The benefits of mentorship extend beyond the operating room. Mentors can provide career guidance, networking opportunities, and support for research and academic pursuits. They inspire trainees to strive for excellence and instill in them a commitment to lifelong learning [75].

In the rapidly evolving field of ear surgery, mentorship is essential for keeping trainees abreast of the latest advancements. Mentors introduce new technologies and techniques, helping trainees integrate these innovations into their surgical practice [76].

Effective mentorship requires a strong commitment from both the mentor and the mentee. Mentors must be dedicated to teaching and nurturing the next generation of surgeons, while mentees must be eager to learn and receptive to feedback. The mentor-mentee relationship is built on trust, respect, and a shared passion for the art and science of ear surgery [77].

Modern Approaches

Modern ear surgery training utilizes technological advancements such as VR simulation and 3D-printed models. These methods offer a safe and effective environment for practicing surgical techniques and improving anatomical understanding. VR simulation provides a realistic experience, allowing trainees to interact with anatomical structures and perform procedures without risk to patients. Three-dimensional-printed models, based on patient-specific imaging data, offer a tangible representation of ear anatomy, facilitating surgical planning and education [78]. 

Three-dimensional models: Three-dimensional-printed models have revolutionized surgical education by offering tangible, customized replicas of patient anatomy generated from high-resolution CT data [79]. These models serve as critical educational benchmarks, allowing trainees to practice manual dexterity and spatial reasoning in a risk-free setting before progressing to live surgical intervention [80]. They bridge the gap between theoretical knowledge and practical application by providing a physical representation of the complex spatial relationships within the temporal bone [81].

A significant modern advancement is the ability to practice on 3D models that replicate specific pathological "ears," such as those with chronic otitis media, cholesteatoma, or congenital malformations [82]. By simulating procedural steps on these patient-specific pathologies, clinicians can anticipate unique anatomical challenges and mitigate potential iatrogenic complications in a controlled environment. This transition from passive observation to active tactile manipulation allows for a steepened learning curve, particularly when dealing with rare or highly variable disease presentations [83].

From a logistical standpoint, 3D-printed models are highly cost-effective and readily accessible compared to traditional specimens. While a single cadaveric session can cost over $2,500, certain 3D-printed training models can be produced or acquired for significantly less, making them a sustainable alternative for high-volume residency programs [84]. Despite these advantages, the accuracy of the models remains dependent on the quality of the initial imaging data, and ongoing research is required to ensure that the texture and consistency of printing materials perfectly replicate the tactile feedback of real human bone [85].

VR simulation: VR simulation has emerged as a groundbreaking technology in surgical education, offering a realistic and immersive training experience that complements traditional methods. By creating a digital environment that mimics the operating room, VR simulation allows trainees to refine their microsurgical skills and practice complex procedures, such as mastoidectomies and stapedotomies, without the risks associated with operating on live patients [86]. These systems provide immediate performance analysis and allow for high-volume repetition of specific surgical steps, which is essential for mastering the spatial constraints of the temporal bone [87].

Despite these benefits, the widespread adoption of VR is often hindered by significant financial barriers and technical limitations. While individual sessions are cost-effective once the system is in place, the initial capital investment is substantial, with acquisition costs reaching approximately $80,000 and annual maintenance fees ranging from $8,000 to $15,000. In comparison, animal models or single-use 3D models present much lower upfront financial burdens for training institutions [88].

Furthermore, a persistent "haptic gap" remains a primary critique of current VR platforms. Digital simulations often fail to fully replicate the nuanced tactile resistance and specific bone-drilling characteristics encountered when working with real biological tissue. While high-resolution graphics and haptic feedback systems enhance engagement, the lack of realistic tactile feedback may limit the realism of the training and affect the transfer of skills to the operating room [89].

Emerging Technologies

Emerging technologies such as exoscopic and robotic surgery are poised to revolutionize ear surgery training by offering enhanced precision, improved ergonomics, and minimally invasive capabilities. Exoscopic surgery, with its magnified and high-definition view of the surgical field, allows for greater accuracy and control in otosurgery procedures. This technology also improves ergonomics for surgeons, reducing physical strain and fatigue. Robotic surgery, while still in its early stages in ear surgery, offers the potential for even greater precision and minimally invasive approaches. Robotic systems, with their miniature instruments and high-definition cameras, can access and manipulate structures within the ear with unparalleled dexterity. These emerging technologies not only enhance surgical precision but also provide a valuable platform for surgical training, allowing trainees to practice complex procedures in a simulated environment and refine their skills before operating on live patients [90]. 

Exoscopic surgery: Exoscopic surgery has emerged as a cutting-edge technology in otology, utilizing high-definition camera systems to project a magnified, 3D view of the surgical field onto high-resolution monitors [91]. This technology provides significant ergonomic benefits by allowing surgeons to maintain a comfortable, upright posture, which reduces the physical strain and neck fatigue commonly associated with prolonged use of traditional operating microscopes [92]. By eliminating the need to hunch over oculars, exoscopes promote a more sustainable surgical practice and reduce the risk of musculoskeletal injuries for the surgeon [93].

From an educational perspective, exoscopes offer a more immersive and interactive training environment than traditional methods [94]. Because the trainee observes the surgical field from the exact perspective of the operating surgeon with the same level of detail, it facilitates a deeper understanding of complex anatomical relationships and surgical techniques. This shared visual experience improves communication within the operating room and allows for real-time guidance, making it a powerful tool for surgical pedagogy [95].

Despite these advantages, the integration of exoscopic surgery into training programs faces practical hurdles, primarily the substantial capital investment required for equipment acquisition and maintenance [96]. Furthermore, there is a clear learning curve associated with transitioning from a direct ocular view to a monitor-based interface, requiring trainees to adapt their hand-eye coordination. Concerns also persist regarding visual fatigue and eye strain during extended procedures, which may necessitate adjustments to traditional surgical workflows to optimize safety and performance [97].

Robotic surgery: Robotic integration within ear surgery, though still in its beginning stages, offers significant promise for enhancing procedural precision and standardizing surgical education [98]. By utilizing miniature instruments and motion-scaling technology, robotic systems can manipulate delicate structures within the middle and inner ear with a degree of dexterity that exceeds the natural capabilities of the human hand. This augmented dexterity is particularly valuable in high-stakes procedures like cochlear implantation, where the goal is to achieve an atraumatic insertion of the electrode array [99].

Beyond clinical applications, robotic platforms serve as sophisticated training tools that provide objective data for skill assessment [100]. The ability to record and analyze surgical movements allows for continuous performance feedback, enabling trainees to identify specific areas for improvement and build confidence in a simulated environment. Robotic systems also hold the potential for developing minimally invasive ear surgery techniques, utilizing smaller incisions and natural orifices to promote faster patient recovery and reduced scarring [101].

However, the widespread adoption of robotics in otologic training is significantly limited by high costs and technical complexity [102]. Establishing a robotic curriculum requires a substantial financial commitment from training institutions, as well as specialized instruction to help trainees master the complex interface. Surgeons and residents must undergo extensive training to adapt their techniques to the robotic system, representing a steep learning curve that must be balanced with traditional hands-on experience to ensure comprehensive surgical proficiency [103].

Discussion

Comparing training modalities

The diverse training modalities available for ear surgery each offer unique advantages that must be balanced against significant logistical and financial constraints. While cadaveric dissection remains the gold standard for haptic realism and anatomical understanding, its utility is increasingly limited by high specimen costs, reaching up to $5,000 per whole-body cadaver, and the lack of dynamic physiological responses such as active bleeding or tissue swelling [104]. Conversely, animal models, such as the ovine model, provide a more cost-effective alternative for refining microsurgical handling, though trainees must engage in "translational calibration" to account for species-specific anatomical discrepancies in temporal bone pneumatization and facial nerve topography [105].

Modern simulation technologies, including VR and 3D printing, have successfully bypassed many ethical and logistical barriers associated with human tissue, yet they introduce a persistent "haptic gap". VR platforms facilitate high-volume repetition and objective performance analysis but require substantial upfront investments of approximately $80,000, alongside annual maintenance fees exceeding $8,000 [106]. Three-dimensional-printed models offer the unique advantage of practicing on patient-specific pathological "ears," but their effectiveness remains contingent on the quality of imaging data and the ability of synthetic materials to replicate the tactile resistance of real bone [107].

Emerging technologies like exoscopic and robotic surgery further expand the training landscape by enhancing visualization and precision. Exoscopes improve ergonomics and facilitate a shared teaching environment, though they may induce visual fatigue and require significant capital for equipment acquisition. Robotic surgery, while offering unparalleled dexterity and motion scaling, presents a steep learning curve and high technical complexity that requires specialized training and a complete adaptation of traditional surgical techniques [108].

Ultimately, the apprenticeship model of mentorship remains the irreplaceable cornerstone of otologic education, synthesizing technical repetition into sound surgical judgment. A mentor provides the tacit knowledge necessary to navigate unexpected intraoperative complications, such as a high-riding jugular bulb or dehiscent facial nerve, which digital algorithms cannot currently codify. The ideal pedagogical approach is therefore a multimodal framework that utilizes simulators to standardize early technical exposure and quantify progression, thereby optimizing patient safety before a trainee enters the high-stakes environment of the operating suite [109].

Despite the comprehensive nature of this review, several limitations must be acknowledged. The transition from simulation-based training to clinical proficiency remains difficult to quantify, as there is a lack of longitudinal studies confirming the direct transfer of skills from high-tech platforms to improved patient outcomes. Furthermore, the rapid pace of technological advancement means that many emerging tools, such as robotic systems, lack the widespread validation and standardized curricula necessary for universal adoption. Finally, the high cost of advanced simulators and exoscopic equipment creates a disparity in training access, potentially limiting these educational benefits to well-funded academic institutions while traditional, specimen-dependent methods remain the only option for many global programs [110].

Advantages and disadvantages

Each training modality for ear surgery presents its own set of advantages and disadvantages that we summarized in Table 1.

**Table 1:** Comparison of Training Modalities in Ear Surgery

Training Modality	Advantages	Disadvantages
Cadaveric dissection	Unparalleled realism and tactile feedback. Deep understanding of ear anatomy. Opportunity to practice on real tissue. Exposure to anatomical variations. Development of surgical skills in a safe environment [64].	Limited availability of specimens. Ethical considerations and costs associated with using cadavers. Lack of dynamic physiological responses. Time-consuming and require specialized facilities [67].
Animal models	More accessible and cost-effective alternative to cadavers. Wider range of training scenarios. Simulation of specific ear pathologies. Opportunity to practice on tissue with closer resemblance to human anatomy [70].	Anatomical differences between species. Ethical considerations regarding animal use [72].
Mentorship	Guidance, support, and personalized instruction from experienced surgeons. Insights into surgical techniques, decision-making, and patient management. Fosters development of surgical judgment and confidence. Provides career guidance and networking opportunities [75].	Effectiveness can vary depending on the mentor's teaching style and the mentee's learning preferences [77].
VR simulation	Safe and immersive environment for practicing complex procedures. Eliminates risk to live patients. No need for cadaveric specimens. Easily updated to incorporate the latest techniques. Provides immediate feedback and performance analysis. Cost-effective and accessible [88].	The cost of VR equipment can be a barrier. Lack of tactile feedback may limit realism [89].
3D-printed models	Tangible and customized representation of patient anatomy. Facilitates surgical planning and preoperative practice. Safe and controlled environment for practicing techniques. Cost-effective and readily accessible. Can simulate various surgical scenarios [84].	Texture and consistency may not perfectly replicate real tissue. Accuracy depends on the quality of imaging data [85].
Exoscopic surgery	Enhances visualization and improves ergonomics. Offers a more immersive training experience. Reduces physical strain and fatigue for surgeons. Facilitates a deeper understanding of surgical techniques and anatomical relationships [92].	The cost of exoscopic equipment can be substantial. Potential for visual fatigue and eye strain. Requires adaptation of surgical techniques [95].
Robotic surgery	Enhanced precision and minimally invasive capabilities. Valuable training platform for complex procedures. Potential for smaller incisions, reduced scarring, and faster recovery times [98].	High cost and technical complexity. Requires specialized training and adaptation of surgical techniques [102].

Future directions

The future of ear surgery training holds exciting possibilities with the continued development of innovative technologies and training approaches that we discussed in Table 2.

**Table 2:** Future Directions in Otologic Surgical Education

Future Direction	Description	Potential Benefits	Challenges
Enhanced simulation	Incorporating haptic feedback and artificial intelligence (AI) algorithms into VR platforms to provide a more immersive and personalized training experience [87].	More realistic and engaging simulations. Personalized feedback and guidance. Improved skill acquisition and retention [31,108].	Developing sophisticated haptic feedback systems and AI algorithms. Ensuring the accuracy and reliability of AI-powered feedback [89].
Augmented reality (AR)	Overlaying digital information, such as 3D anatomical structures, onto the real-world surgical field [90].	Real-time guidance during procedures. Enhanced visualization of anatomical structures. Improved precision and safety [30].	Developing robust AR systems that seamlessly integrate with surgical workflows. Ensuring accurate registration and tracking of digital information [95].
Hybrid simulation	Combining VR and AR technologies with traditional methods, such as cadaveric dissection and mentorship [62].	Comprehensive training experience that leverages the strengths of different modalities. Optimized learning process [110].	Integrating different training modalities effectively. Balancing the use of technology with traditional hands-on experience [109].
Personalized training programs	Utilizing AI-powered platforms to analyze individual trainee performance and tailor training programs to specific needs and learning styles [86].	Optimized learning process. Accelerated skill acquisition. Improved training efficiency [31].	Developing sophisticated AI algorithms that can accurately assess trainee performance and personalize training programs [104].
Global collaboration	Facilitating collaboration and knowledge sharing among surgeons and trainees worldwide through online platforms and virtual reality environments [107].	Democratized access to surgical training. Dissemination of best practices. Global exchange of knowledge and expertise [89].	Addressing logistical and technological challenges associated with global collaboration. Ensuring equitable access to training resources [73].
Focus on human factors	Greater emphasis on non-technical skills, such as communication, teamwork, and decision-making [65].	Holistic training approach that prepares trainees for the cognitive and interpersonal challenges of surgery. Improved patient safety and outcomes [13].	Developing effective training methods for non-technical skills. Integrating human factors training with technical skills development [109].
Ethical considerations	Addressing the ethical implications of new training modalities, such as patient safety, data privacy, and equitable access [63].	Responsible and ethical implementation of new technologies. Promotion of trust and transparency in surgical training [110].	Developing ethical guidelines and frameworks for the use of emerging technologies in surgical training. Ensuring equitable access to training resources [90].

## Conclusions

Ear surgery training demands a comprehensive and adaptable approach that respects the intricate anatomy and functional sensitivity of the temporal bone. Traditional methods, including cadaveric dissection and mentorship, continue to provide the fundamental basis for anatomical knowledge and the development of surgical judgment. These time-tested approaches are now being effectively augmented by modern simulations that offer a safe and repeatable environment for refining technical skills without the limitations of specimen availability or risks to live patients.

The integration of emerging technologies, such as exoscopic and robotic systems, holds potential for further enhancing surgical precision and ergonomic safety. These tools allow for greater magnification and tremor filtration, which are essential for the microscopic scale of otologic interventions. However, their widespread adoption remains contingent upon future validation, the reduction of capital costs, and the development of structured, evidence-based curricula that can effectively bridge the gap between simulation and the operating room.

In summary, the future of otologic education lies in the balanced integration of high-fidelity simulation, AI-assisted assessment, and traditional mentorship. By embracing innovation while acknowledging the technical and financial hurdles of new modalities, training programs can ensure that surgeons acquire the necessary proficiency in a standardized manner. This multimodal evolution is essential for maintaining the highest standards of patient safety and surgical excellence in an increasingly complex technological landscape.

## References

[1] Wiet GJ, Sørensen MS, Andersen SA (2017). Otologic skills training. Otolaryngol Clin North Am.

[2] Aussedat C, Venail F, Marx M, Boullaud L, Bakhos D (2022). Training in temporal bone drilling. Eur Ann Otorhinolaryngol Head Neck Dis.

[3] Chien WW, da Cruz MJ, Francis HW (2021). Validation of a 3D-printed human temporal bone model for otology surgical skill training. World J Otorhinolaryngol Head Neck Surg.

[4] Riojas KE, Labadie RF (2020). Robotic ear surgery. Otolaryngol Clin North Am.

[5] Hudise JY, Mojiri ME, Shawish AM (2024). The role of virtual reality in advancing surgical training in otolaryngology: A systematic review. Cureus.

[6] Lähde S, Hirsi Y, Salmi M, Mäkitie A, Sinkkonen ST (2024). Integration of 3D-printed middle ear models and middle ear prostheses in otosurgical training. BMC Med Educ.

[7] Arora A, Lau LY, Awad Z, Darzi A, Singh A, Tolley N (2014). Virtual reality simulation training in otolaryngology. Int J Surg.

[8] Luers JC, Hüttenbrink KB (2016). Surgical anatomy and pathology of the middle ear. J Anat.

[9] Moneta LB, Quintanilla-Dieck L (2017). Embryology and anatomy of the ear. Oper Tech Otolayngol Head Neck Surg.

[10] Hendry C, Farley A, McLafferty E (2012). Anatomy and physiology of the senses. Nurs Stand.

[11] Chatterjee S, Das S, Ganguly K, Mandal D (2024). Advancements in robotic surgery: Innovations, challenges and future prospects. J Robot Surg.

[12] Tarabichi O, Jensen M, Hansen MR (2021). Advances in hearing preservation in cochlear implant surgery. Curr Opin Otolaryngol Head Neck Surg.

[13] Sharp MC, MacfArlane R, Hardy DG, Jones SE, Baguley DM, Moffat DA (2005). Team working to improve outcome in vestibular schwannoma surgery. Br J Neurosurg.

[14] Ribeiro DS, Jotz GP, Sousa NC (2021). Image-guided temporal bone dissection course. Int Arch Otorhinolaryngol.

[15] Erdem S, Fazliogullari Z, Ural A, Karabulut AK, Unver Dogan N (2022). External ear anatomy and variations in neonates. Congenit Anom (Kyoto).

[16] Anschuetz L, Presutti L, Marchioni D, Bonali M, Wimmer W, Villari D, Caversaccio M (2018). Discovering middle ear anatomy by transcanal endoscopic ear surgery: A dissection manual. J Vis Exp.

[17] Marchioni D, Rubini A, Soloperto D (2021). Endoscopic ear surgery: Redefining middle ear anatomy and physiology. Otolaryngol Clin North Am.

[18] Driver EC, Kelley MW (2020). Development of the cochlea. Development.

[19] Mackowetzky K, Yoon KH, Mackowetzky EJ, Waskiewicz AJ (2021). Development and evolution of the vestibular apparatuses of the inner ear. J Anat.

[20] Schick B, Dlugaiczyk J (2013). Surgery of the ear and the lateral skull base: Pitfalls and complications. GMS Curr Top Otorhinolaryngol Head Neck Surg.

[21] Antonelli PJ (2018). Prevention and management of complications in otosclerosis surgery. Otolaryngol Clin North Am.

[22] Orlando VR, Cruz OL (2024). Postoperative complications in cochlear implant surgery and their possible risk factors. Braz J Otorhinolaryngol.

[23] Blebea CM, Ujvary LP, Necula V (2022). Current concepts and future trends in increasing the benefits of cochlear implantation: A narrative review. Medicina (Kaunas).

[24] Milenkovic I, Schiefer U, Ebenhoch R, Ungewiss J (2020). [Anatomy and physiology of the auditory pathway]. Ophthalmologe.

[25] Young AS, Rosengren SM, Welgampola MS (2018). Disorders of the inner-ear balance organs and their pathways. Handb Clin Neurol.

[26] Vankatova L, Hsieh JW, Daskalou D, Senn P (2022). Mechanically evoked tinnitus after cochlear implantation with preservation of residual hearing. Cochlear Implants Int.

[27] Bartels LJ (1991). Facial nerve and medially invasive petrous bone cholesteatomas. Ann Otol Rhinol Laryngol.

[28] Selesnick SH, Lynn-Macrae AG (2001). The incidence of facial nerve dehiscence at surgery for cholesteatoma. Otol Neurotol.

[29] Chi FL, Gao Z (2024). [The path to advancement in endoscopic ear surgery]. Zhonghua Yi Xue Za Zhi.

[30] Gaffuri M, di Lullo AM, Trecca EM (2023). High-definition 3D exoscope in pediatric otorhinolaryngology: A systematic literature review. J Clin Med.

[31] Bhutta MF (2016). A review of simulation platforms in surgery of the temporal bone. Clin Otolaryngol.

[32] Kuru I, Maier H, Müller M, Lenarz T, Lueth TC (2016). A 3D-printed functioning anatomical human middle ear model. Hear Res.

[33] Mitchell S, Coulson C (2017). Endoscopic ear surgery: A hot topic?. J Laryngol Otol.

[34] Nogueira JF, de Sousa Lobo Ferreira Querido R, Gonçalves da Silva Leite J, Cabral da Costa T (2021). Future of endoscopic ear surgery. Otolaryngol Clin North Am.

[35] Stern Shavit S, Sharma RK, Chern A, Golub JS (2021). Pearls and pitfalls in endoscopic ear surgery. Otolaryngol Clin North Am.

[36] Sharma MO, Pareek Y, Sehra R, Jat KS (2022). Hearing outcome in ossiculoplasty with autologous incus and teflon prosthesis in chronic otitis media: A comparative study. Indian J Otolaryngol Head Neck Surg.

[37] Bhavana K (2017). Our experience of treating wide spectrum of external ear canal atresia of different etiologies in pediatric patients. Indian J Otolaryngol Head Neck Surg.

[38] Kennedy KL, Lin JW (2023). Mastoidectomy. StatPearls [Internet].

[39] Prasad SC, Piras G, Piccirillo E, Taibah A, Russo A, He J, Sanna M (2016). Surgical strategy and facial nerve outcomes in petrous bone cholesteatoma. Audiol Neurootol.

[40] Nadol JB Jr (2006). Revision mastoidectomy. Otolaryngol Clin North Am.

[41] Dornhoffer JL (1999). Surgical modification of the difficult mastoid cavity. Otolaryngol Head Neck Surg.

[42] Geerse S, Bost TJ, Allagul S, de Wolf MJ, Ebbens FA, van Spronsen E (2020). Hearing and hearing rehabilitation after obliteration of troublesome mastoid cavities. Eur Arch Otorhinolaryngol.

[43] Lou ZC, Lou ZH, Zhang QP (2012). Traumatic tympanic membrane perforations: A study of etiology and factors affecting outcome. Am J Otolaryngol.

[44] Dornhoffer JL (2006). Cartilage tympanoplasty. Otolaryngol Clin North Am.

[45] Bartel R, Cruellas F, Hamdan M, Gonzalez-Compta X, Cisa E, Domenech I, Manos M (2018). Hearing results after type III tympanoplasty: Incus transposition versus PORP. A systematic review. Acta Otolaryngol.

[46] Agarwal D, Chandra M (2023). Hearing assessment after total annular excision tympanoplasty- Our institutional experience. Indian J Otolaryngol Head Neck Surg.

[47] Mudhol RS, Naragund AI, Shruthi VS (2013). Ossiculoplasty: Revisited. Indian J Otolaryngol Head Neck Surg.

[48] Dornhoffer JL, Gardner E (2001). Prognostic factors in ossiculoplasty: A statistical staging system. Otol Neurotol.

[49] De Vos C, Gersdorff M, Gérard JM (2007). Prognostic factors in ossiculoplasty. Otol Neurotol.

[50] Cox MD, Page JC, Trinidade A, Dornhoffer JL (2017). Long-term complications and surgical failures after ossiculoplasty. Otol Neurotol.

[51] Isaacson B, Hunter JB, Rivas A (2018). Endoscopic stapes surgery. Otolaryngol Clin North Am.

[52] Cheng HC, Agrawal SK, Parnes LS (2018). Stapedectomy versus stapedotomy. Otolaryngol Clin North Am.

[53] Nitta Y, Sano H, Furuki S, Yamashita T (2023). Long-term outcomes of stapes surgery. Auris Nasus Larynx.

[54] Yancey KL, Manzoor NF, Rivas A (2021). Endoscopic stapes surgery: Pearls and pitfalls. Otolaryngol Clin North Am.

[55] Luryi AL, Schettino A, Bojrab DI, Babu SC, Michaelides EM, Bojrab DI 2nd, Schutt CA (2021). Hearing outcomes and complications in stapes surgery for otosclerosis performed under general or local anesthesia. Otolaryngol Head Neck Surg.

[56] Moses LE, Friedmann DR (2021). Cochlear implant indications: A review of third-party payers' policies for standard and expanded indications. Cochlear Implants Int.

[57] Naples JG, Ruckenstein MJ (2020). Cochlear implant. Otolaryngol Clin North Am.

[58] Abari J, Heuninck E, Al Saadi M, Topsakal V (2023). True keyhole cochlear implant surgery. Am J Otolaryngol.

[59] Topsakal V, Agrawal S, Atlas M (2022). Minimally traumatic cochlear implant surgery: Expert opinion in 2010 and 2020. J Pers Med.

[60] Dindelegan MG, Blebea C, Perde-Schrepler M, Buzoianu AD, Maniu AA (2022). Recent advances and future research directions for hearing loss treatment based on nanoparticles. J Nanomater.

[61] Gumus B, İncesulu AS, Kaya E, Kezban Gurbuz M, Ozgur Pınarbaslı M (2021). Analysis of cochlear implant revision surgeries. Eur Arch Otorhinolaryngol.

[62] Rak K, Kaulitz S, Voelker J (2024). Online training for hearing implant surgery: A new approach to otological training. HNO.

[63] Michelson JD, Manning L (2008). Competency assessment in simulation-based procedural education. Am J Surg.

[64] Morris DP, Luff DA, Hargreaves SP, Rothera MP (1998). Bones of contention. The supply of temporal bones for dissection: The legalities, problems and solutions. J Laryngol Otol.

[65] Smith JM (2020). Surgeon coaching: Why and how. J Pediatr Orthop.

[66] Fennessy BG, O'Sullivan P (2009). Establishing a temporal bone laboratory: Considerations for ENT specialist training. Ir J Med Sci.

[67] Naik SM, Naik MS, Bains NK (2014). Cadaveric temporal bone dissection: Is it obsolete today?. Int Arch Otorhinolaryngol.

[68] Fermi M, Chiari F, Mattioli F (2022). Surgical training on ex vivo ovine model in otolaryngology head and neck surgery: A comprehensive review. Int J Environ Res Public Health.

[69] Blebea CM, Dindelegan MG, Ujvary LP, Susman S, Maniu AA, Cosgarea M (2022). Relevant anatomical details in rats regarding otologic research. Int J Morphol.

[70] Stan C, Ujvary PL, Blebea C (2024). Hand motion analysis using accelerometer-based sensors and sheep's head model for basic training in functional endoscopic sinus surgery. Cureus.

[71] Gurr A, Pearson MD, Dazert S (2011). [Lambs' temporal bone anatomy under didactic aspects]. Braz J Otorhinolaryngol.

[72] Sudhakara Rao M, Chandrasekhara Rao K, Raja Lakshmi C, Satish Chandra T, Murthy PS (2019). Suitable alternative for human cadaver temporal bone dissection: Comparative micro ear anatomy of cattle, pig and sheep with human. Indian J Otolaryngol Head Neck Surg.

[73] Rajendran L, Jones D, Brar S (2022). Junior Mentorship Program (JuMP) start in surgery-Implications on trainee success. J Surg Educ.

[74] Chun R, Jabbour N, Balakrishnan K (2016). Education on, exposure to, and management of vascular anomalies during otolaryngology residency and pediatric otolaryngology fellowship. JAMA Otolaryngol Head Neck Surg.

[75] Macafee DA (2008). Is there a role for mentoring in surgical specialty training?. Med Teach.

[76] Silver CM, Yuce TK, Clarke CN (2024). Disparities in mentorship and implications for US surgical resident education and wellness. JAMA Surg.

[77] Goosmann M, Williams AM, Grewal J, Patel J, Jones L, Yaremchuk KL (2023). The importance of female mentors and safety in the workplace to female otolaryngology applicants when creating rank lists. Ear Nose Throat J.

[78] Kashikar TS, Kerwin TF, Moberly AC, Wiet GJ (2019). A review of simulation applications in temporal bone surgery. Laryngoscope Investig Otolaryngol.

[79] Pugliese L, Marconi S, Negrello E (2018). The clinical use of 3D printing in surgery. Updates Surg.

[80] Gadaleta DJ, Huang D, Rankin N (2020). 3D printed temporal bone as a tool for otologic surgery simulation. Am J Otolaryngol.

[81] Hochman JB, Rhodes C, Wong D, Kraut J, Pisa J, Unger B (2015). Comparison of cadaveric and isomorphic three-dimensional printed models in temporal bone education. Laryngoscope.

[82] Mooney MA, Cavallo C, Zhou JJ (2020). Three-dimensional printed models for lateral skull base surgical training: Anatomy and simulation of the transtemporal approaches. Oper Neurosurg.

[83] Bakhos D, Velut S, Robier A, Al zahrani M, Lescanne E (2010). Three-dimensional modeling of the temporal bone for surgical training. Otol Neurotol.

[84] Razavi C, Galaiya D, Vafaee S, Yin R, Carey JP, Taylor RH, Creighton FX (2021). Three dimensional printing of a low-cost middle-ear training model for surgical management of otosclerosis. Laryngoscope Investig Otolaryngol.

[85] Wiet GJ, Stredney D, Powell K, Hittle B, Kerwin T (2016). Integration of high-resolution data for temporal bone surgical simulations. Int J Comput Assist Radiol Surg.

[86] Matwala K, Shakir T, Bhan C, Chand M (2024). The surgical metaverse. Cir Esp (Engl Ed).

[87] Sieber DM, Andersen SA, Sørensen MS, Mikkelsen PT (2021). OpenEar image data enables case variation in high fidelity virtual reality ear surgery. Otol Neurotol.

[88] Locketz GD, Lui JT, Chan S, Salisbury K, Dort JC, Youngblood P, Blevins NH (2017). Anatomy-specific virtual reality simulation in temporal bone dissection: Perceived utility and impact on surgeon confidence. Otolaryngol Head Neck Surg.

[89] Seddon I, Rosenberg E, Houston SK 3rd (2023). Future of virtual education and telementoring. Curr Opin Ophthalmol.

[90] Geiger JD, Hirschl RB (2015). Innovation in surgical technology and techniques: Challenges and ethical issues. Semin Pediatr Surg.

[91] Tan SH, Kulasegarah J, Prepageran N (2023). Experience with 2D exoscope system for bilateral simultaneous cochlear implant surgery in the era of the COVID-19 pandemic. Indian J Otolaryngol Head Neck Surg.

[92] Wierzbicka M, Szyfter W, Greczka G, Gawęcki W (2021). Otosurgery with the high-definition three-dimensional (3D) exoscope: Advantages and disadvantages. J Clin Med.

[93] Lin ME, Zhou S, Kakeheta S, Ito T, Shibata SB (2024). Ergonomics of various modalities for ear surgery. OTO Open.

[94] Tu NC, Doerfer K, Costeloe A, Sioshansi PC, Babu S (2024). Educational benefit of the three-dimensional exoscope versus operating microscope in otologic surgery. Otol Neurotol.

[95] Kanzaki S, Takahashi S, Toda M, Yoshida K, Ogawa K (2021). Pros and cons of the exoscope for otologic surgery. Surg Innov.

[96] Ferlito S, La Mantia I, Caruso S (2022). High definition three-dimensional exoscope (VITOM 3D) in E.N.T. surgery: A systematic review of current experience. J Clin Med.

[97] Chiang H, Ledbetter L, Kaylie DM (2022). Systematic review of otologic and neurotologic surgery using the 3-dimensional exoscope. Otol Neurotol Open.

[98] Lane T (2018). A short history of robotic surgery. Ann R Coll Surg Engl.

[99] Dahroug B, Tamadazte B, Weber S, Tavernier L, Andreff N (2018). Review on otological robotic systems: Toward microrobot-assisted cholesteatoma surgery. IEEE Rev Biomed Eng.

[100] Zagzoog N, Yang VX (2018). State of robotic mastoidectomy: Literature review. World Neurosurg.

[101] Kazmitcheff G, Nguyen Y, Miroir M (2014). Middle-ear microsurgery simulation to improve new robotic procedures. Biomed Res Int.

[102] Panara K, Shahal D, Mittal R, Eshraghi AA (2021). Robotics for cochlear implantation surgery: Challenges and opportunities. Otol Neurotol.

[103] Zhu TS, Godse N, Clayburgh DR, Duvvuri U (2022). Assessing the learning curve associated with a novel flexible robot in the pre-clinical and clinical setting. Surg Endosc.

[104] Halliday GR, Sudworth P, Counter PR, Tustin H (2026). Simulators in endoscopic ear surgery: A systematic review of models, validation and educational utility. J Laryngol Otol.

[105] Mowry SE, Hansen MR (2014). Resident participation in cadaveric temporal bone dissection correlates with improved performance on a standardized skill assessment instrument. Otol Neurotol.

[106] Stan C, Blebea C, Tănase MI (2024). Exploring the ovine anatomy: A comprehensive study of the sheep's head for basic training in functional endoscopic sinus surgery. Cureus.

[107] Vaghaiwalla T, Gyawali S, Jayaram A (2023). Academic global surgery: Creating opportunities, equity, and diversity. Ann Glob Health.

[108] Stan C, Vesa D, Tănase MI (2023). Can non-virtual reality simulation improve surgical training in endoscopic sinus surgery? A literature review. Adv Med Educ Pract.

[109] Lee SY, Jeong B, Lee W, Park MK (2024). Feasibility of easy to use and inexpensive three-dimensional printed educational model of temporal bone: Practiced without drilling. J Med Educ Curric Dev.

[110] Calloni T, Roumy LG, Cinalli MA (2022). Exoscope as a teaching tool: A narrative review of the literature. Front Surg.

